# Clinical Outcomes and Live Birth Rate Resulted From Microdissection Testicular Sperm Extraction With ICSI-IVF in Non-Obstructive Azoospermia: A Single-Center Cohort Study

**DOI:** 10.3389/fendo.2022.893679

**Published:** 2022-06-23

**Authors:** Yu Lan, Haiyan Zheng, Xin Fu, Tianwen Peng, Chen Liao, Jianan Liu, Min Liu, Geng An

**Affiliations:** ^1^ Department of Obstetrics and Gynecology, Center for Reproductive Medicine, Key Laboratory for Major Obstetric Diseases of Guangdong Province, The Third Affiliated Hospital of Guangzhou Medical University, Guangzhou, China; ^2^ Center for Reproductive Medicine, The First Affiliated Hospital, Zhejiang University School of Medicine, Hangzhou, China

**Keywords:** intracytoplasmic sperm injection, microdissection testicular sperm extraction, non-obstructive azoospermia, testicular sperm, pregnancy rate, live birth rate

## Abstract

**Background:**

Most of data available in the literature reported the sperm retrieval rate and limited intracytoplasmic sperm injection (ICSI) results of microdissection testicular sperm extraction (micro-TESE) in non-obstructive azoospermia (NOA) patients with different etiologies. Unfortunately, there is currently a lack of comprehensive data to guide clinicians in conducting comprehensive consultations with NOA patients.

**Objectives:**

To obtain more comprehensive evidence-based data and clinical outcomes for better consultation of NOA patients who opted to undergo micro-TESE combined with ICSI-IVF

**Methods:**

It was a retrospective study involved 968 NOA patients underwent micro-TESE during January 2015 to December 2019. Embryological, clinical, and live birth outcomes were demonstrated comprehensively and three kinds of stratification analyses were performed based on ICSI-IVF cycles using frozen and fresh sperm, different etiologies of NOA and various amounts of sperm retrieved.

**Results:**

The sperm retrieval rate was 44.6%, and ICSI was performed in 299 couples leading to 150 clinical pregnancies and 140 live-birth deliveries. The clinical pregnancy rate (CPR) was 50.17%, and the cumulative live birth rate (LBR) was 46.82%, and the low birth defects rate was 1.43%. No significant difference was observed about cumulative LBR in frozen sperm group and fresh sperm group (47.5% vs 42.9%, *P>*0.05). NOA patients with AZFc microdeletions had the lowest rate of a high-score embryo on day 3 (4.4%, *P*<0.05) and the lowest cumulative LBR (19.4%, *P*<0.05). NOA patients with lower sperm count (having fewer than 20 sperms retrieved) had significantly lower cumulative LBR than those with higher sperm count (having more than 20 sperms retrieved) (28.1% vs 51.9%, *P*<0.05).

**Conclusions:**

For those NOA patients who stepped in ICSI-IVF cycles, the cumulative LBR was 46.82%. No significant difference was indicated in the LBR between ICSI-IVF cycles using frozen or fresh testicular sperm. Compared to other etiologies, NOA caused by AZFc microdeletions have the poorest embryological and clinical outcomes. Patients with less testicular sperm retrieved have poorer embryological and clinical outcomes.

## 1 Introduction

Non-obstructive azoospermia (NOA) is the most severe form of male infertility and is characterized by the testis’s inability to produce mature sperm, and NOA accounts for 60% of all patients with azoospermia (1). Based on the different causes of non-obstructive azoospermia, non-genetic etiologies include cryptorchidism, heat exposure, infections, and chemoradiotherapy. The most common genetic causes are Y chromosome microdeletions and chromosomal abnormalities (2). However, most NOA patients have the unknown cause of their azoospermia (3). Because of spermatogenesis, couples with NOA used donor sperm or opted for adoption to have children before current sperm retrieval methods (4).

The development of intracytoplasmic sperm injection (ICSI) in 1992 provided a novel opportunity for azoospermia patients to become fathers (5). Sperm obtained through testicular sperm extraction (TESE) was first performed on NOA patients for ICSI in 1995 (6). TESE-ICSI then became a routine procedure to treat NOA patients. Studies reported that the sperm retrieval rate (SRR) of TESE in the NOA population was approximately 30 - 50%. However, these studies’ selective bias makes numbers controversial (7, 8). Subsequently, various surgical techniques, such as multiple testicular biopsies and fine-needle aspiration, were used to improve SRR and reduce complications in NOA patients, but each has its limitations (9). In the past 20 years, microdissection testicular sperm extraction (micro-TESE) has gradually become a popular surgical technique with a high SRR and low tissue loss (10, 11). However, most of the previous studies were focus on SRR, and few could provide comprehensive follow-up data on ICSI-IVF outcomes of NOA patients, explicitly focusing on the use of fresh or frozen sperm, cumulative pregnancy rate (PR), and live birth rate (LBR), not alone birth defect rate (12, 13).

Our goal was to obtain more comprehensive evidence-based data and clinical outcomes for better preoperative consultation and counseling of NOA patients who opted to undergo micro-TESE combined with ICSI-IVF. This retrospective study aimed to analyze the effects of frozen or fresh sperm, different etiologies, and retrieved sperm quantity on ICSI-IVF outcomes after micro-TESE.

## 2 Materials and Methods

### 2.1 Patients Selection

**Figure 1:**
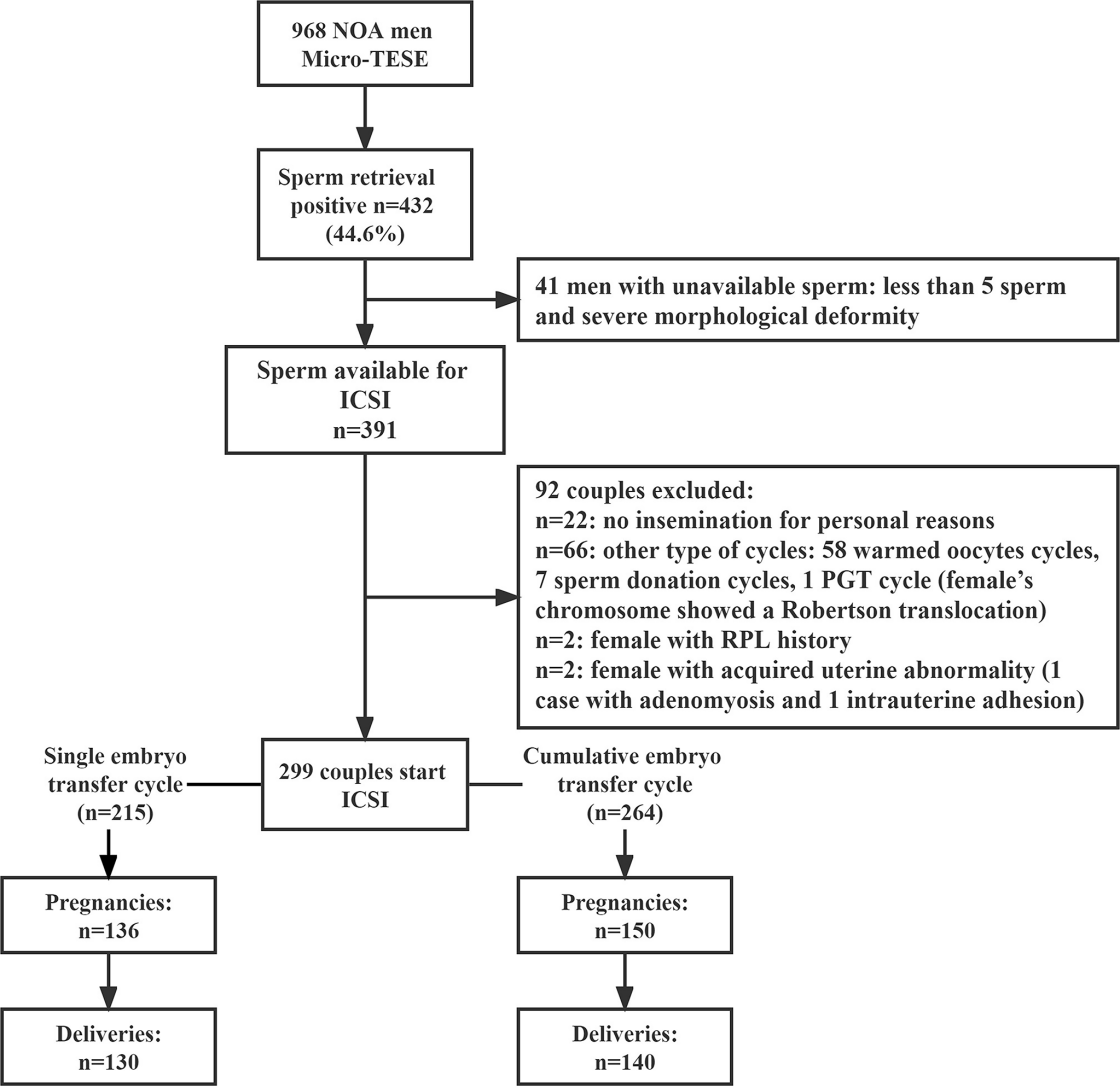
Schematic overview of the study.

This retrospective study included 968 men with NOA who underwent micro-TESE in the Reproductive Medicine Center of Third Affiliated Hospital of Guangzhou Medical University between January 2015 and December 2019 (**Figure 1**). All semen samples were centrifuged at 3,000 g for 30 minutes to confirm azoospermia and on at least three separate occasions. NOA patients underwent a complete clinical evaluation to determine the etiology of azoospermia, including clinical history, physical examination, testicular ultrasound, evaluation of sex hormone levels [follicle stimulating hormone (FSH), luteinizing hormone (LH) testosterone (T), estradiol (E2), and prolactin (PRL)], karyotyping and Y chromosome microdeletion analysis.

If azoospermia combined with testicular atrophy and abnormality of sex hormone, the etiological diagnosis process was as follows: ①NOA caused by Klinefelter’s syndrome or AZF microdeletions was diagnosed if there was karyotyping or Y chromosome microdeletion abnormalities. ②NOA caused by orchitis was diagnosed if there was a history of orchitis after mumps infection in adolescence or adulthood. ③NOA caused by cryptorchidism was diagnosed if there was a combination of ultrasound diagnosis or previous history of cryptorchidism (especially surgical history). ④Idiopathic NOA was diagnosed by excluding known causes of NOA. When testicular volume, texture and endocrine examination were normal, testicular pathological biopsy was required to confirm whether there was spermatogenesis disorder. All patients underwent micro-TESE were routinely performed with testicular biopsy during surgery to further confirm spermatogenesis dysfunction. Patients with a history of micro-TESE, a history of radiation and chemotherapy, anejaculation, or hypogonadotropic hypogonadism were excluded. The same surgeon (GA) performed all micro-TESE procedures. This study was approved by the Ethics Committee of the Third Affiliated Hospital of Guangzhou Medical University (reference number 2017-055) and was carried out in accordance with the Helsinki Declaration. Due to the retrospective nature, informed consent was not required, and patients` data were used anonymously.

Before micro-TESE surgery was conducted, complete clinical consulting was performed regarding the treatment protocol selection and male factors that may influence the outcome of ICSI-IVF treatment. In addition, the female’s ovarian reserve would be assessed in detail before the eventual decision on the conduction of micro-TESE surgery to reduce the risk of cycle cancellation due to the female factors. Advanced age (≥40 years old, 6 cases), diminished ovarian reserve (AMH ≤ 1ng/ml), or poor ovarian response (≤4 oocytes retrieved) were all defined as risk factors for adverse outcomes, and patients would be informed of high risk of ICSI-IVF failure.

All NOA couples were offered the option to receive fresh or frozen testicular sperm for ICSI cycles before surgical sperm retrieval and be informed of their respective advantages and disadvantages. For those using frozen sperm, controlled ovarian stimulation (COS) will be initiated after the sperm is successfully assessed for cryopreservation. For those using fresh sperm, they would be informed that using fresh sperm can minimize the operation step of sperm before injection which may reduce energy loss and the risk of operation error, especially when very few sperm are retrieved. However, the simultaneous ICSI cycles of fresh sperm and egg collection may result in unnecessary COS, oocytes retrieval, and passive egg freezing due to sperm retrieval failure or unimplemented surgery.

### 2.2 Micro-TESE

A single surgeon (GA) performed the micro-TESE surgical procedures as described in the previous literature (14), a few minor and technical modifications, including the use of saline instead of Ringer’s solution as the rinsing fluid and suturing the albuginea testis with a 5-0 polypropylene suture instead of 6-0. Instead of the surgeon grinding the testicular tissue on the operating table, an embryologist grind the testicular tissue and microscopically search sperm on a bench setting near the operating table (15). If no sperm was identified in one testis, micro-TESE of the contralateral testis was performed immediately.

### 2.3 Evaluation of Sperm Quality

After testicular tissue was retrieved from the testis and transferred to embryologist, spermatozoa was further processed to be released. Procedures were performed at room temperature as follows: wash testicular tissue with 1 ml G-MOPS-Plus fluid (Vitrolife, Sweden) to remove the red blood cells; Move testicular tissue into a new petri dish and add 1 ml new G-MOPS-Plus fluid and grind the tissue into tiny patches by using microscopic forceps to release spermatozoa. Then, the grinded testicular tissue homogenate was evaluated under high power magnification (200×).

All sperm were counted out and recorded if the amount was less than 100. When the total sperm amount was more than 100, it will be recorded as “>100”. We defined the lower sperm count (LSC) group as having fewer than 20 sperms and defined the higher sperm count (HSC) group as more than 20 sperms. Motile spermatozoa rate was determined (motile spermatozoa rate = motile sperm/total sperm × 100%). If the total number of sperm was greater than 100, the number of motile sperm was quantified in 100 sperm. The sperm deformity was not be record because it could not be assessed accurately under 200× magnification.

For statistical analysis, there was another method for the evaluation of sperm amount before and after cryopreservation (as section 4 below).

### 2.4 Testicular Tissue Suspension Cryopreservation and Thawed

After testicular tissue homogenate was evaluated and transferred to the embryology laboratory, a freezing process was immediately performed. First, transfer all fluid and tissues into a 15 ml centrifuge tube; Add 1 ml fresh G-MOPS-Plus fluid to the petri dish to wash additional tissue from the dish and transfer into the 15-ml tube; Incubate at room temperature for five minutes; Aspirate the supernatant to a fresh new 15-ml centrifuge tube; Add 1ml G-MOPS-Plus fluid to the pellet, and transfer the supernatant to the fresh new 15-ml tube. Centrifuge the tube at 400g for 10 minutes, and remove all the supernatant (remaining 0.5ml). Sucking 50μl out to count under the microscope and recorded the amount of sperm in each high magnification field, and recycle these 50μl. Next, the freezing procedures were performed as follows: add freezing medium (Test York Buffer with Gentamicin Sulfate) (Irvine Scientific, USA) and G-MOPS-Plus in a 1:1 ratio into a pellet and resuspend, aliquot resuspended pellet into cryovials; and place at t 4°C for 30 minutes; Suspend the mixture prior to placing in liquid nitrogen for 1 hour. Transfer the cryovials into a liquid nitrogen container for long-term cryopreservation. The thawing process was performed as follows. Remove the cryovial from the liquid nitrogen container, place it at room temperature for 10 minutes; Transfer the thawed liquid to a fresh 15ml centrifuge tube; Add drops of IM washing buffer (Vitrolife, Sweden) to the tube, and mix gently. Centrifuge at 400g for 10 minutes, and then remove the supernatant. Resuspend the pellet with 1-2 ml IM washing buffer. After the second wash, resuspend the sperm pellet with 50-100 μl G-MOPS-Plus fluid. The count and motility of thawed sperm were reassessed.

### 2.5 Ovarian Stimulation and Oocytes Retrieval

In couples who had testicular sperm was retrieved and frozen, or in couples undergoing a synchronous micro-TESE-ICSI procedure, female partners underwent ovarian stimulation using recombinant FSH or hMG combined GnRH antagonists or GnRH-a (16). Oocyte–cumulus complexes were recovered 36h after administering 5000 or 10 000 IU of hCG.

### 2.6 ICSI Procedure, Embryo Culture, and Transfer

For couples who had sperm cryopreserved, sperm were thawed only when females had oocytes retrieved. For couples who underwent synchronous micro-TESE-ICSI treatment, oocytes were vitrified if no sperm retrieved. Next, ICSI-IVF and fertilization assessment were performed as previously described by Liu (16). Fertilization rates were expressed as the percentage of oocytes with two distinct pronuclei per injected metaphase II oocytes. Embryos were scored by their morphological appearance according to the Society for Assisted Reproductive Technology scoring system (17). Normally cleaving embryos with ≥5 cells and ≤20% fragmentation were considered eligible for transfer. Up to two embryos were transferred into the uterine cavity on day 3 (preferred) or day 5 after injection. The remaining embryos were frozen directly for the next thawed transfer cycles. All patients had completed at least one embryo transfer by the end of follow-up.

### 2.7 Pregnancy Follow-Up

Pregnancy was diagnosed by elevated serum hCG levels (≥25IU/L) 14 days after embryo transfer. Clinical pregnancy was defined as a visible gestational sac at transvaginal ultrasound 4-5 weeks after embryo transfer. Pregnancy loss was defined as the loss of a clinical pregnancy before 28 weeks of gestation. Live birth was defined as the birth of at least one living child, irrespective of gestation duration. The cumulative pregnancy or live birth was defined as clinical pregnancy or at least one live-born baby resulting from an ICSI-IVF initiated cycle.

### 2.8 Statistical Analysis

Statistical analyses were performed with SPSS statistical software for Windows, version 22.0 (SPSS, Chicago, IL, USA). All data were normally distributed and continuous variables were expressed as mean ± SD. The ‘Student’s t-test was used for comparison of continuous variables. One-way ANOVA was used to assess outcomes among more than two groups. Chi-squared (χ2) or Fisher’s exact test was used for proportions. Differences were considered statistically significant when the *p*-value was <0.05.

## 3 Results

### 3.1 Micro-TESE and Sperm Recovery

A total of 968 patients with NOA underwent micro-TESE, and 432 had sperm retrieved (sperm retrieval rate, SRR=44.6%). A total of 299 patients were with sperm retrieved were consecutively enrolled in ICSI treatment (**Figure 1**). The SRR showed significantly different among the different etiologies, including orchitis (81.2%), Klinefelter Syndrome (KS) (43.6%), Y chromosome azoospermia factor c (AZFc) microdeletions (68.6%), cryptorchidism (62.4%), and idiopathic (31.1%) (**Supplementary Table 1**) (*p*<0.01).

Sperm quality and quantity were evaluated under a microscope before freezing and after thawing, and assessment included a total of sperm count, percentage of motile sperm, and the percentage of abnormal sperm (teratozoospermia). No significant differences were found in the total sperm count and motility of testicular sperm between the different groups (**Supplementary Table 2**) (*p*>0.05).

### 3.2 Outcomes of ICSI-IVF

ICSI-IVF was performed in 299 couples, leading to 136 clinical pregnancies and 130 live-birth deliveries in the first embryo transfer cycle, and 150 clinical pregnancies and 140 live-birth deliveries in cumulative embryo transfer cycle (**Figure 1**).

Among those NOA patients with micro-TESE-ICSI, the fertilization rate was 69.85%, the 2PN rate was 61.49%, the day 3 utilization rate was 28.35%, the rate of high-score embryo on day 3 was 12.75%, and the rate of unavailable embryos was 11.7%. In the first embryo transfer cycle, the clinical pregnancy rate (CPR) was 45.48% and the LBR was 43.49%. In the cumulative embryo transfer cycle, CPR was as high as 56.82% and LBR was 53.03% in those with available embryos, and 50.17% of CPR and 46.82% of LBR in those undergoing ICSI cycles, and 34.72% of CPR and 32.41% in those who have successful sperm retrieval (**Table 1**, **2**).

**Table 1:** Basic characteristics and ICSI outcomes of the NOA patients with sperm retrieved by Micro-TESE.

Variable	Parameters
**Male**	
Age (yr.)	31.52 ± 4.77
BMI (kg/m^2^)	24.49 ± 3.46
Left Testicular volume (ml)	7.01 ± 3.77
Right Testicular volume (ml)	6.97 ± 3.82
Hormone profile	
FSH (IU/L)	19.42 ± 10.89
LH (IU/L)	9.46 ± 5.58
T (ng/ml)	10.73 ± 6.92
**Female**	
Age (yr.)	29.10 ± 4.05
AMH (ng/ml)	5.02 ± 3.83
BMI (kg/m^2^)	21.94 ± 3.39
Infertility type	
Primary (%)	83.9 (251/299)
Secondary (%)	16.1 (48/299)
**COS-ICSI outcomes**	
Days of ovarian stimulation	10.61 ± 2.20
Total gonadotropin dose (IU)	1888.93 ± 920.67
Oestradiol level on hCG trigger day (pmol/L)	11139.50 ± 4606.68
Progesterone level on hCG trigger day (nmol/L)	2.41 ± 1.33
The endometrial thickness on hCG trigger day (mm)	10.52 ± 2.02
Number of oocytes retrieved	14.38 ± 7.24
Number of MII oocytes for ICSI	10.96 ± 7.97
Fertilization rate (%)	69.85 (2289/3277)
2PN rate (%)	61.49 (2015/3277)
2PN cleavage rate (%)	81.30 (1861/2289)
Day 3 utilization rate of MII eggs for ICSI (%)^1^	28.35 (929/3277)
Rate of high-score embryo on day 3 (%)^2^	12.75 (257/2015)
No. of cycles without available embryos/ICSI cycles (%)	11.7 (35/299)
Number of available embryos on day 3	3.11 ± 2.80
Number of high-score embryos on day 3	0.86 ± 1.14

Data are expressed as mean ± SD unless indicated otherwise.

^1^Computational formula: number of available embryos on day3/number of MII oocytes for ICSI.

^2^Computational formula: number of high-score embryos on day 3/number of 2PN.

ICSI, intracytoplasmic sperm injection; NOA, non-obstructive azoospermia; Micro-TESE, microdissection testicular sperm extraction; BMI, body mass index; FSH, follicle-stimulating hormone; LH, luteinizing hormone; T, testosterone; AMH, anti-mullerian hormone; COS, controlled ovarian stimulation; hCG, human choroinic gonadotrophin; PN, Primary nucleus.

**Table 2:** Embryo transfer and live birth outcomes.

Variable	NOA couples
**1^st^ embryo transfer cycle**	
No. of embryos transferred	1.55 ± 0.50
Clinical pregnancy rate (%)	45.48 (136/299)
Live birth rate (%)	43.49 (130/299)
**Cumulative Pregnancy**	
No. of patients with clinical pregnancy/undergoing ICSI cycles with available embryos (%)	56.82 (150/264)
No. of patients with clinical pregnancy/undergoing ICSI cycles (%)	50.17 (150/299)
No. of patients with clinical pregnancy/successful sperm retrieval (%)	34.72 (150/432)
**Cumulative Live birth**	
No. live birth/clinical pregnancy (%)	93.33 (140/150)
Singleton (%)	87.14 (122/140)
Twins (%)	12.86 (18/140)
No. live birth/undergoing ICSI cycles with available embryos (%)	53.03 (140/264)
No. live birth/undergoing ICSI cycles (%)	46.82 (140/299)
No. live birth/successful sperm retrieval (%)	32.41 (140/432)
No. premature infants/live birth (%)	18.57 (26/140)
No. cesarean delivery/live birth (%)	31.43 (44/140)
No. birth defects/live birth (%)^#^	1.43 (2/140)
**Gender**	
Male (%)	39.24 (62/158)
Female (%)	60.76 (96/158)
**Birth weight (g)**	2981.27 ± 534.65
Singleton (n =122)	3174.18 ± 419.13
Twins (n =18, 36 babies)	2327.50 ± 324.95
**Height (cm)**	48.65 ± 3.13
Singleton (n =122)	49.66 ± 2.23
Twins (n =18, 36 babies)	45.19 ± 3.32

Data are expressed as mean ± SD unless indicated otherwise.

^#^One case is a cardiovascular malformation, and the other is a cleft lip and palate.

ICSI, intracytoplasmic sperm injection; NOA, non-obstructive azoospermia.

According to the follow-up data, there were 26 premature infants in 140 newborns and two cases of birth defects recorded. One case is a cardiovascular malformation, and the other is a cleft lip and palate. (**Table 2**).

First of all, a stratification analysis based on using frozen or fresh testicular sperm was performed. All couples undergoing the ICSI-IVF cycle were divided into frozen sperm group and fresh sperm group (257 cases with frozen sperm *vs.* 42 cases with fresh sperm). The fertilization rate, 2 primary nucleus (PN) rate, numbers of available and high-score embryos on day 3 were comparable between these two groups. There were no significant differences in PR and LBR between these two groups no matter in the first embryo transfer cycle (PR: 45.9% vs. 42.9%, LBR: 44.0% vs. 40.5%, *p*>0.05) or cumulative embryo transfer cycles (PR: 51.0% vs. 45.2%, LBR: 47.5 vs. 42.9%, *p*>0.05). Besides, the percentage of patients with no available embryo was low in both frozen and fresh sperm groups and was not significantly different between the two groups (11.3% vs. 14.3%). Follow-up data showed that compared to the fresh sperm group, singleton newborns of frozen sperm group have higher height (49.84 ± 2.04cm *vs*. 48.50 ± 3.03cm, *p*<0.05) (**Table 3**).

**Table 3:** Comparison of the ICSI outcomes between frozen and fresh sperm.

	Frozen sperm (n=257)	Fresh sperm (n=42)	*P*-value
**Age (yr)**			
Male	31.58 ± 4.91	31.10 ± 3.86	0.540
Female	29.01 ± 4.07	29.64 ± 3.94	0.350
**COS-ICSI outcomes**	/	/	/
Days of ovarian stimulation	10.62 ± 2.29	10.50 ± 1.55	0.738
Total gonadotropin dose (IU)	1914.55 ± 959.68	1732.14 ± 617.26	0.235
Estradiol level on HCG trigger day (pmol/L)	10789.34 ± 4528.86	13282.10 ± 4550.46	0.001^*^
Progesterone level on HCG trigger day (nmol/L)	2.35 ± 1.30	2.77 ± 1.50	0.059
The endometrial thickness on HCG trigger day (mm)	10.56 ± 2.08	10.29 ± 1.66	0.417
Number of oocytes retrieved	13.87 ± 7.13	17.50 ± 7.23	0.002^*^
Number of MII oocytes for ICSI	11.02 ± 8.29	10.62 ± 5.73	0.766
Fertilization rate (%)	70.1 (1985/2831)	68.2 (304/446)	0.403
			0.516^#^
2PN rate (%)	61.8 (1750/2831)	59.4 (265/446)	0.333
			0.684^#^
2PN cleavage rate (%)	91.9 (1609/1750)	95.1 (252/265)	0.072
			0.447^#^
Number of available embryos on day 3	3.09 ± 2.94	2.86 ± 2.25	0.625
			0.074^#^
Number of high-score embryos on day 3	0.84 ± 1.15	0.98 ± 1.05	0.474
			0.899^#^
Day 3 utilization rate of MII eggs for ICSI (%)^1^	28.7 (812/2831)	26.2 (117/446)	0.286
			0.899^#^
Rate of high-score embryo on day 3 (%)^2^	12.3 (216/1750)	15.5 (41/265)	0.16
			0.062^#^
No. of cycles without available embryos/ICSI cycles (%)	11.3 (29/257)	14.3 (6/42)	0.575
**1^st^ embryo transfer cycle**			
No. of embryos transferred	1.56 ± 0.51	1.61 ± 0.55	0.533
Clinical pregnancy rate (%)	45.9 (118/257)	42.9 (18/42)	0.712
			0.452^#^
Live birth rate (%)	44.0 (113/257)	40.5 (17/42)	0.672
			0.499^#^
**One ICSI cycle**			
Cumulative pregnancy rate per women (%)	51.0 (131/257)	45.2 (19/42)	0.491
Cumulative live birth rate per women (%)	47.5 (122/257)	42.9 (18/42)	0.579
No. live birth/clinical pregnancy (%)	93.1 (122/131)	94.7 (18/19)	0.793
Singleton (%)	86.9 (106/122)	88.9 (16/18)	0.813
Twins (%)	13.1 (16/122)	11.1 (2/18)	
Birth defect (%)	1.6 (2/122)	0 (0/18)	0.584
** Gender**	/	/	0.941
Male (%)	39.1 (54/138)	40.0 (8/20)	/
Female (%)	60.9 (84/138)	60.0 (12/20)	/
** Birth weight (g)**			
Singleton	3194.67 ± 405.16	3038.44 ± 495.32	0.166
Twins	2302.50 ± 331.11	2527.50 ± 197.55	0.196
** Height (cm)**			
Singleton	49.84 ± 2.04	48.50 ± 3.03	0.024^*^
Twins	44.91 ± 3.40	47.50 ± 1.00	0.143

Data are expressed as mean ± SD unless indicated otherwise

1 Computational formula: number of available embryos on day3 / number of MII oocytes for ICSI. 2 Computational formula: number of high-score embryos on day 3 / number of 2PN. *Significantly different.

# P value after correcting the estradiol level on HCG trigger day.

ICSI, intracytoplasmic sperm injection; COS, controlled ovarian stimulation.

P value after corrected the estradiol level on HCG trigger day.

Next, a stratification analysis based on different etiologies of NOA was performed. The results showed a statistical difference in day 3 utilization rate of MII eggs for ICSI among each group, with the lowest percentage observed in patients with Y chromosome AZFc microdeletions (22.3%), followed by KS (24.4%), cryptorchidism (27.2%), orchitis (30.8%), and was observed to be the highest in patients with idiopathic NOA (31.7%). The percentage of patients with no available embryo was significantly different among the five etiologies of NOA, with the highest percentage in patients with Y chromosome AZFc microdeletions (25.8%) (**Supplementary Figure 1**). Besides, the lowest rate of the high-score embryo on day 3 (4.4%), lowest cumulative CPR (22.6%), lowest cumulative LBR with successful sperm retrieval (19.4%), and the highest rate of premature birth (50%) were observed in patients with Y chromosome AZFc microdeletions (*p<*0.05) (**Table 4**).

**Table 4:** Comparison of the ICSI outcomes of NOA patients with different etiologies.

	Idiopathic(n=112)	Orchitis(n=37)	Klinefelter`s Syndrome (n=80)	AZFc Microdeletions(n=31)	Cryptorchidism(n=39)	*P*-value
Male`s age (yr.)	32.48 ± 5.45	32.22 ± 4.42	30.05 ± 4.22	31.03 ± 3.93	31.46 ± 4.01	0.009^*^
Female`s age (yr.)	29.50 ± 4.06	30.68 ± 4.30	27.94 ± 4.16	28.42 ± 3.16	29.38 ± 3.60	0.006^*^
Male’s FSH (IU/L)	16.14 ± 10.16	22.16 ± 10.56	24.00 ± 11.29	19.72 ± 9.46	16.59 ± 9.76	0.000^*^
No. of MII oocytes for ICSI	11.79 ± 10.98	11.95 ± 6.91	10.69 ± 5.45	8.97 ± 4.78	9.79 ± 3.71	0.329
No. of available embryos on day3	3.73 ± 3.50	3.68 ± 3.00	2.61 ± 1.85	2.00 ± 2.07	2.67 ± 1.88	0.003^*^
No. of high-score embryos on day 3	1.06 ± 1.36	1.05 ± 1.15	0.76 ± 0.93	0.26 ± 0.51	0.77 ± 0.99	0.006^*^
Day 3 utilization rate of MII eggs for ICSI %^1^	31.7 (418/1320)	30.8 (136/442)	24.4 (209/855)	22.3 (62/278)	27.2 (104/382)	0.000^*^
Rate of high-score embryo on day 3%^2^	14.9 (119/801)	14.7 (39/266)	11.7 (61/522)	4.4 (8/180)	12.2 (30/246)	0.003^*^
No. of cycles without available embryos/ICSI cycles (%)	13.4 (15/112)	5.4 (2/37)	8.8 (7/80)	25.8 (8/31)	7.7 (3/39)	0.062
Sperm quantity (%)	/	/	/	/	/	0.011^*^
≤20 sperms	15.2 (17/112)	13.5 (5/37)	31.2 (25/80)	35.5 (11/31)	15.4 (6/39)	/
>20 sperms	84.8 (95/112)	86.5 (32/37)	68.8 (55/80)	64.5 (20/31)	84.6 (33/39)	/
Sperm motility rate (%, mean ± SD)	13.69 ± 9.96	11.46 ± 9.93	12.53 ± 11.23	10.23 ± 9.88	16.07 ± 10.71	0.118
**Pregnancy**	/	/	/	/	/	/
No. of patients with clinical pregnancy/undergoing ICSI cycles with available embryos (%)	58.8 (57/97)	62.9 (22/35)	57.5 (42/73)	30.4 (7/23)	61.1 (22/36)	0.113
No. of patients with clinical pregnancy/undergoing ICSI cycles (%)	50.9 (57/112)	59.5 (22/37)	52.5 (42/80)	22.6 (7/31)	56.4 (22/39)	0.021^*^
No. of patients with clinical pregnancy/successful sperm retrieval (%)	42.0 (68/162)	63.8 (30/47)	46.6 (54/116)	14.3 (7/49)	46.6 (27/58)	0.000^*^
**Live birth**	/	/	/	/	/	/
No. live birth/clinical pregnancy (%)	91.2 (52/57)	95.5 (21/22)	95.2 (40/42)	85.7 (6/7)	95.5 (21/22)	0.805
Singleton (%)	86.5 (45/52)	90.5 (19/21)	92.5 (37/40)	66.7 (4/6)	81.0 (17/21)	0.378
Twins (%)	13.5 (7/52)	9.5 (2/21)	7.5 (3/40)	33.3 (2/6)	19.0 (4/21)	0.095
No. live birth/undergoing ICSI cycles with available embryos (%)	53.6 (52/97)	60.0 (21/35)	54.8 (40/73)	26.1 (6/23)	58.3 (21/36)	
No. live birth/undergoing ICSI cycles (%)	46.4 (52/112)	56.8 (21/37)	50.0 (40/80)	19.4 (6/31)	53.8 (21/39)	0.018^*^
No. live birth/successful sperm retrieval (%)	37.0 (60/162)	57.4 (27/47)	44.8 (52/116)	12.2 (6/49)	44.8 (26/58)	0.000^*^
No. premature infants/live birth (%)	15.4 (8/52)	14.3 (3/21)	25.0 (10/40)	50.0 (3/6)	9.5 (2/21)	0.150
No. cesarean delivery/live birth (%)	36.5 (19/52)	42.9 (9/21)	22.5 (9/40)	33.3 (2/6)	23.8 (5/21)	0.412
No. birth defects/live birth (%)	1.9 (1/52)	0	2.5 (1/40)	0	0	0.892
Gender	/	/	/	/	/	0.906
Male (%)	42.4 (25/59)	34.8 (8/23)	34.9 (15/43)	37.5 (3/8)	44.0 (11/25)	/
Female (%)	57.6 (34/59)	65.2 (15/23)	65.1 (28/43)	62.5 (5/8)	56.0 (14/25)	/
Birth weighBirth weight (g)	/	/	/	/	/	/
Singleton (n =122)	3254.44 ± 396.96	3178.68 ± 398.13	3063.51 ± 478.25	2995.00 ± 528.93	3239.71 ± 301.16	0.252
Twins (n =18, 36 babies)	2156.43 ± 419.94	2495.00 ± 82.26	2459.17 ± 265.87	2411.25 ± 87.21	2402.50 ± 209.61	0.152
Height (cm)	/	/	/	/	/	/
Singleton (n =122)	49.84 ± 2.04	49.53 ± 2.82	49.41 ± 2.42	48.00 ± 2.45	50.29 ± 1.21	0.352
Twins (n =18, 36 babies)	44.36 ± 3.78	48.00 ± 3.16	45.83 ± 3.37	44.25 ± 0.96	45.25 ± 3.01	0.381

Data are expressed as mean ± SD unless indicated otherwise.

^1^ and ^2^, the definitions see **Table 1**.

^*^ Significantly different.

Furthermore, a stratification analysis based on sperm quantity was performed. All patients who successfully entered the ICSI cycle were separated into lower sperm count (LSC, ≤20 sperms) and higher sperm count (HSC, >20 sperms) groups. No significant difference was observed in male or female age between the two groups (*p*>0.05). NOA patients with LSC showed higher FSH levels, and their female partners underwent more partial oocyte freezing (23.4%, *p*<0.05). In all initial ICSI cycles (including cycles without available embryos on day 3) in the PP set, couples with LSC had significantly lower 2PN cleavage rate, lower day 3 utilization rate of MII eggs for ICSI, a lower rate of the high-score embryo on day 3, lower cumulative CPR (29.7% vs. 55.7%, *p*<0.05), lower cumulative LBR (28.1% vs. 51.9%, *p*<0.05) and a significantly higher risk of no available embryos on day 3 compared with couples with HSC (*p*<0.05). Additionally, significantly lower cumulative CPR (37.5% vs. 56.5%, *p*<0.05) was observed in couples with LSC than couples with HSC when patients who had no available embryos were excluded (**Table 5**).

**Table 5:** Comparison of the ICSI outcomes of NOA patients with different sperm quantity.

	Lower sperm count(n =64)	Higher sperm count(n =235)	*P*-value
Male’s age (y)	31.11 ± 4.56	31.63 ± 4.83	0.444
Female’s age (y)	28.97 ± 4.30	29.14 ± 3.99	0.770
Male’s FSH (IU/L)	21.89 ± 12.62	18.75 ± 10.29	0.041^*^
No. of MII oocytes for ICSI	15.09 ± 6.76	14.19 ± 7.37	0.376
No. of available embryos on day3	2.14 ± 1.92	3.37 ± 2.94	0.002^*^
No. of high-score embryos on day 3	0.58 ± 0.87	0.94 ± 1.19	0.025^*^
Day 3 utilization rate of MII eggs for ICSI %^1^	9.67 ± 5.05	11.31 ± 8.58	0.145
Rate of high-score embryo on day 3%^2^	9.5 (37/390)	13.5 (220/1625)	0.031^*^
No. of partial oocytes freezing cycles/OPU cycles (%)	23.4 (15/64)	11.9 (28/235)	0.020^*^
No. of patients without available embryos/ICSI cycles (%)	25.0 (16/64)	8.1 (19/235)	0.000^*^
**Pregnancy**			
No. of patients with clinical pregnancy/undergoing ICSI cycles with available embryos (%)	39.6 (19/48)	60.6 (131/216)	0.008^*^
No. of patients with clinical pregnancy/undergoing ICSI cycles (%)	29.7 (19/64)	55.7 (131/235)	0.000^*^
No. of patients with clinical pregnancy/successful sperm retrieval (%)	19.0 (24/126)	52.9 (162/306)	0.000^*^
**Live birth**			
No. live birth/clinical pregnancy (%)	94.7 (18/19)	93.1 (122/131)	0.793
Singleton (%)	100.0 (18/18)	85.2 (104/122)	0.081
Twins (%)	0 (0/18)	14.8 (18/122)	
No. live birth/undergoing ICSI cycles with available embryos (%)	37.5 (18/48)	56.5 (122/216)	0.017^*^
No. live birth/undergoing ICSI cycles (%)	28.1 (18/64)	51.9 (122/235)	0.001^*^
No. live birth/successful sperm retrieval (%)	17.5 (22/126)	48.7 (149/306)	0.000^*^
No. premature infants/live birth (%)	5.6 (1/18)	22.1 (27/122)	0.101
No. cesarean delivery/live birth (%)	22.2 (4/18)	32.8 (40/122)	0.367
No. birth defects/live birth (%)	5.6 (1/18)	0.8 (1/122)	0.114
Gender	/	/	0.974
Male (%)	38.9(7/18)	39.3(55/140)	/
Female (%)	61.1(11/18)	60.7(85/140)	/
Birth weight (g)	/	/	/
Singleton (n =122)	3073.33 ± 461.11	3191.63 ± 411.33	0.271
Twins (n =18, 36 babies)	0	2327.50 ± 324.95	/
Height (cm)	/	/	/
Singleton (n =122)	48.94 ± 2.82	49.79 ± 2.10	0.139
Twins (n =18, 36 babies)	0	45.19 ± 3.32	/

Data are expressed as mean ± SD unless indicated otherwise; ^1^ and ^2^, the definitions see **Table 1**.

^*^ Significantly different.

## 4 Discussion

This study’s main strength is comprehensively analyzing clinical outcomes for NOA patients undergoing micro-TESE and ICSI-IVF cycle which minimally or barely reported in the past literature. A total of 299 NOA patients who had stepped in ICSI cycles were evaluated, which is the largest retrospective study with sample size at present. In addition, female factors that may affect the ICSI-IVF outcomes were excluded, including recurrent pregnancy loss, abnormal karyotype, known uterine anomalies, and adenomyosis, improving extrapolation of our results.

### 4.1 Micro-TESE-ICSI has Considerable Live Birth Outcomes

According to our study, the fertilization rate was as high as 69.85%, the day 3 utilization rate of MII eggs for ICSI was 28.35%, the cumulative CPR was 50.17%, and the cumulative LBR was 46.82% which high up to the same as conventional ICSI-IVF as reported (18). Besides, there were no stillbirths, pregnancy complications, or neonatal complications in our study. Overall, the opportunity for a NOA patient to be a biological father is 14.5% (140/968) for NOA patients before he accept micro-TESE treatment. With successful sperm retrieving, this opportunity rise up to 32.41%, and once his sperm be assessed as suitable for ICSI cycle, the chance rise up to 46.82%. It is worth to mention that only about 11.7% patients may not be able to obtain available embryos according to one ICSI cycle. However, poor outcomes were observed in this study in those couples who has female risk factors (with advanced female age ≥40 years old; AMH ≤ 1ng/ml; ≤4 oocytes retrieved) for ICSI-IVF. All of these patients treated with ICSI lead to one live-birth delivery in 6 patients aged ≥40 years, three live-birth deliveries in 16 diminished ovarian reserve patients, and 15 live-birth deliveries in 29 poor ovarian response patients (data not included in the results section). The female-factor combined with male-factor assessment refined our clinical consultation on ICSI treatment for couples with NOA. Those detailed data will help physicians provide with sufficient counseling and avoid patients` over-high expectations of treatment. Thus, this study provides a tremendous clinical basis for reproductive medicine specialists to better understand the clinical outcome of micro-TESE combined with ICSI-IVF cycle, which allows for more comprehensive preoperative clinical consultation and preparations.

### 4.2 Similar Live Birth Outcomes Were Observed in ICSI-IVF Cycles Using Frozen or Fresh Testicular Sperm

Not all NOA patients have access to fresh sperm, and couples who want to synchronize the ICSI-IVF cycles with fresh sperm and oocyte have to face the risk of oocyte freezing and sperm donation-IVF. Therefore, sperm cryopreservation before ICSI may be more reasonable and reduce unnecessary risks for females. Cryopreservation of testicular sperm has long been used in assisted reproductive technology (19). Previous studies confirmed significant differences between fresh and frozen sperm by ejaculation, regardless of total sperm count, motility, or morphology of sperm (20); however, the differences contributed little to the outcome of ICSI cycles (21). Due to controversial evaluations with NOA patients, ICSI-IVF outcomes are controversial because of different cryopreservation methods (12, 22).

Our study reported an improved laboratory technique of testicular tissue suspension cryopreservation in NOA patients after sperm retrieval from micro-TESE. We established a technique and method to assess the quantity and quality of sperm, which is simple, reliable, and can be easily used as a conventional assessment method, even for too extremely few spermatozoa patients. We found no difference in the average number and the percentage of motile spermatozoa between frozen-thawed sperm and fresh sperm. At the same time, frozen-thawed sperm with ICSI resulted in a similar fertilization rate (70.1% vs. 68.2%, p=0.403) and day 3 utilization rate of oocytes (28.7% vs. 26.2%, p=0.286) compared with fresh sperm.

According to previous researches, the clinical pregnancy rate between fresh sperm and frozen sperm remains controversial. Park reported that patients with frozen spermatozoa had significantly higher pregnancy and implantation rates than fresh sperm (23). Madureira showed that the fertilization rate and clinical pregnancy rate were higher in fresh sperm from non-mosaic KS patients by TESE (24). A systematic review and meta-analysis revealed that in men with NOA showed that the ICSI-IVF outcome was not affected by whether the retrieved testicular sperm is fresh or frozen (25). Nevertheless, cumulative pregnancy or cumulative live birth was not mentioned in any of the above studies, especially the cumulative live birth. After comparing the outcomes of ICSI-IVF cycles between fresh and frozen-thawed spermatozoa, we found that there were no significant differences in all laboratory parameters, not only fertilization rate and day 3 oocytes utilization rate we mentioned above, but also cleavage rate, rate of the high-score embryo, and the ratio of patients who had no available embryos. The main results we found are consistent with previous studies (12, 21). Then, we conducted a further analysis of clinical outcomes after embryo transfer, including the first embryo transfer cycle and cumulative embryo transfer cycles. Similarly, CPR, LBR, newborns` parameters showed no significant differences between the fresh and frozen groups. Therefore, it could be concluded that using frozen testicular sperm is as effective and safe as fresh testicular sperm in NOA patients with micro-TESE.

### 4.3 Relatively Poor ICSI Outcomes Were Observed in NOA Patients With Y Chromosome AZFc Microdeletions

Few studies provided a detailed analysis of ICSI-IVF outcomes according to the different pathological types and etiologies of NOA patients (12, 26). Madureira demonstrated no significant differences in ICSI-IVF outcomes between different histopathological subsets in NOA (24). However, in our study, there were significant differences in day 3 utilization rate of MII eggs for ICSI-IVF cycle among NOA patients with different etiologies (idiopathic= 31.7%, orchitis= 30.8%, cryptorchidism = 27.2%, KS= 24.4%, and lowest in Y chromosome AZFc microdeletions= 22.3%, p<0.05). Liu reported that fertilization competent, viable embryo rate, and pregnancy rate of spermatozoa retrieved from men with Y chromosome AZFc chromosome deletions were similar to men without it (27). Our results showed that lower day 3 oocytes utilization rate and high-score embryo rate and lower cumulative CPR and cumulative LBR were observed in patients with Y chromosome AZFc microdeletion, which are similar to Zhang`s report but with less heterogeneity, because the objects we included were all testicular sperm by micro-TESE (28). These results are also consistent with the finding reported by Van and the primary function of the AZFc region in the Y chromosome is involvement in spermatozoa quality or function than in spermatogenesis (29).

### 4.4 Relatively Poor ICSI Outcomes Were Observed in NOA Patients With Less Sperm Found

Compared to whom got more sperm (>20 approximately), NOA patients with fewer sperm (≤20 approximately) were detected with significantly higher serum FSH level, lower oocytes utilization rate, lower high-score embryo rate, and a higher ratio of cycles without available embryos. Some studies reported that sperm from NOA patients have aneuploidy, mosaicism, and DNA damage that contribute to decreased clinical outcomes (30). Similarly, in this study, a significantly lower clinical pregnancy rate and live birth rate were observed as expected in patients with fewer sperm. These results provide us with a better understanding of treatment outcomes for patients with different laboratory findings after testicular tissue processing.

### 4.5 Limitations

However, our study still has limitations, including the sample size of the fresh sperm group was not large enough, the need to supplement more follow-up data on live births, and patient selection bias. In the future, multicenter data and randomized controlled trials are needed to determine clinical predictors of successful outcomes for NOA couples.

In conclusion, for those NOA patients who stepped in ICSI-IVF cycles, the cumulative LBR was 46.82%. No significant difference was observed in LBR between ICSI-IVF cycles using frozen or fresh testicular sperm. NOA patients with AZFc microdeletions had the lowest rate of the high-score embryo on day 3 and the lowest cumulative CPR. NOA patients with lower sperm count had significantly lower cumulative LBR than those with higher sperm count.

## Data Availability Statement

The original contributions presented in the study are included in the article/**Supplementary Material**. Further inquiries can be directed to the corresponding author.

## Ethics Statement

The studies involving human participants were reviewed and approved by the Ethics Committee of the Third Affiliated Hospital of Guangzhou Medical University (reference number 2017-055). Written informed consent was not required for this study, in accordance with the local legislation and institutional requirements.

## Author Contributions

The contributions of all authors were as follow: Conceptualization, Data curation, Writing-original draft: GA. Statistical analysis: HZ. Data collection: YL, XF, TP, CL, JL, and ML. All authors have contributed to critical discussion, reviewed the final version of the manuscript and approved it for publication.

## Funding

This study was funded by the National Key R&D Plan (grant nos. 2019YFE0109500), the National Natural Science Foundation of China (grant nos. 82171589), the Guangdong Natural Science Foundation (grant nos. 2019A1515011439) and the Guangzhou Health Science and Technology Project (grant no. 20201A011093).

## Conflict of Interest

The authors declare that the research was conducted in the absence of any commercial or financial relationships that could be construed as a potential conflict of interest.

## Publisher’s Note

All claims expressed in this article are solely those of the authors and do not necessarily represent those of their affiliated organizations, or those of the publisher, the editors and the reviewers. Any product that may be evaluated in this article, or claim that may be made by its manufacturer, is not guaranteed or endorsed by the publisher.

## Supplementary Material

Supplementary Figure 1 Available embryos of ICSI-IVF in NOA patients with different etiologies.
