# Supplementary material for: Clinical Outcomes and Live Birth Rate Resulted From Microdissection Testicular Sperm Extraction With ICSI-IVF in Non-Obstructive Azoospermia: A Single-Center Cohort Study
Source: Front Endocrinol (Lausanne). 2022 Jun 23;13:893679. doi: 10.3389/fendo.2022.893679 (PMC9259991; doi:10.3389/fendo.2022.893679)
Supplement: Supplementary file 2 [file Table_1.docx]

**Supplemental Table S1 SRR in all micro-TESE patients and etiological composition of NOA patients**

| **Etiologies** | **SRR (%)^*^** | **ICSI cycles of PP set** | |
| --- | --- | --- | --- |
|  |  | **Frozen sperm (%) ^#^**  **(n=257)** | **Fresh sperm (%) ^#^**  **(n=42)** |
| Idiopathic | 31.1(144/463) | 42.0 (108) | 9.5 (4) |
| Orchitis | 81.2(56/69) | 10.9 (28) | 21.4 (9) |
| [Klinefelter](https://pubmed.ncbi.nlm.nih.gov/29493939/?from_term=KS+syndrome&from_pos=3)`s syndrome | 43.6(105/241) | 22.2 (57) | 54.8 (23) |
| AZFc microdeletions | 68.6(59/86) | 10.5 (27) | 9.5 (4) |
| Cryptorchidism | 62.4(68/109) | 14.4 (37) | 4.8 (2) |
| Total | 44.6% (432/968) | / | / |

*Data are expressed as n (%).

^*^ Significantly different between five etiologies, *P* = 0.000.

**^#^** Significantly different between five etiologies, *P* = 0.000.

Abbreviation: SRR, sperm retrieval rate.
