# Supplementary material for: Clinical Outcomes and Live Birth Rate Resulted From Microdissection Testicular Sperm Extraction With ICSI-IVF in Non-Obstructive Azoospermia: A Single-Center Cohort Study
Source: Front Endocrinol (Lausanne). 2022 Jun 23;13:893679. doi: 10.3389/fendo.2022.893679 (PMC9259991; doi:10.3389/fendo.2022.893679)
Supplement: Supplementary file 3 [file Table_2.docx]

**Supplemental Table S2** **Evaluation of sperm quality in testicular tissue suspension**

|  | Fresh sperm  (n=42) | Frozen-thawed sperm  (n=257) | | | *P*-Value 2 |
| --- | --- | --- | --- | --- | --- |
|  |  | **Before** | **After** | ***P*-Value 1** |  |
| Count of sperm  (mean±SD/HPF) | 7.17±5.51 | 8.24±7.11 | 7.33±6.52 | 0.572 | 0.170 |
| Motile spermatozoa (%) | 12.61±12.55 | 13.13±10.10 | 11.69±9.92 | 0.103 | 0.768 |

Data are expressed as mean ± SD unless indicated otherwise.

*P*-value 1: comparison between frozen-thawed sperm (before) and frozen-thawed sperm (after).

*P*-value 2: comparison between fresh sperm and frozen-thawed sperm (before).

Abbreviation: HPF, high power field
